# Unconsummated marriage among Chinese couples: a retrospective study

**DOI:** 10.1093/sexmed/qfac003

**Published:** 2023-01-12

**Authors:** Yu Xi, Tingting Xia, Elena Colonnello, Chunlin Wang, Yufen Lai, Yan Zhang

**Affiliations:** Department of Infertility and Sexual Medicine, The Third Affiliated Hospital of Sun Yat-sen University, Guangzhou, China; Center of Reproductive Medicine, The Third Affiliated Hospital of Sun Yat-sen University, Guangzhou, China; Department of Experimental Medicine, Section of Medical Pathophysiology, Food Science and Endocrinology, Sapienza University of Rome, Rome, Italy; Department of Systems Medicine, University of Tor Vergata, Rome, Italy; Department of Infertility and Sexual Medicine, The Third Affiliated Hospital of Sun Yat-sen University, Guangzhou, China; Department of Infertility and Sexual Medicine, The Third Affiliated Hospital of Sun Yat-sen University, Guangzhou, China; Department of Infertility and Sexual Medicine, The Third Affiliated Hospital of Sun Yat-sen University, Guangzhou, China

**Keywords:** unconsummated marriage, erectile dysfunction, female sexual dysfunction, vaginismus, dyspareunia

## Abstract

**Background:**

Unconsummated marriage (UCM) is a significant problem among couples who are unable to achieve successful sexual intercourse and penovaginal penetration, and the etiology and clinical characteristics of UCM in Chinese couples remain unknown.

**Aim:**

In a retrospective analysis of patients with UCM, we investigated clinical characteristics and treatment outcomes among Chinese couples with UCM.

**Methods:**

During the period from January 2019 to May 2021, we examined 127 consecutive couples with unconsummated marriage. The couples were evaluated separately by andrologists and gynecologists, and combined treatments were conducted by therapists.

**Outcomes:**

We calculated the distribution of etiologies of UCM in Chinese couples.

**Results:**

Among the couples whose data were evaluated, 93 couples visited the andrologist first and 34 couples visited the gynecologist first. The most common complaints associated with sexual dysfunction were erectile dysfunction (ED) in male patients and vaginismus and dyspareunia in female patients. Unconsummated marriage among Chinese couples was caused primarily by female factors (55.8%). With couple-oriented treatment conducted by sexual therapists, the success rate was 67.7%.

**Clinical Translation:**

If a couple is diagnosed with UCM, both the husband and wife should be treated individually receive guidance from a sex therapist toward successful sexual intercourse.

**Strengths and Limitations:**

This is to our knowledge the first report regarding the etiology of UCM in Chinese couples. Here we report our routine diagnostic and therapeutic workups. However, we were not able to perform hormonal and imaging studies of the female partners. Moreover, patients presenting with UCM who visited our department without a partner were not included in the statistics.

**Conclusions:**

Unconsummated marriage among Chinese couples may be caused byfactors affecting both the husband and wife or the husband and wife individually; however, factors affecting women are the predominant causes of UCM. Lack of knowledge about sex-related issues, as well as cultural beliefs, play an important role. A preliminary evaluation by an andrologist and a gynecologist, followed by couple treatment conducted by the sex therapist, is highly `recommended to treat UCM effectively.

## Introduction

Unconsummated marriage (UCM) is defined as the failure of a couple to perform successful sexual intercourse at the beginning of marriage,^1^^,^^2^ particularly in the first few nights.^3^ When this occurs, the first sexual intercourse may also be delayed for years,^4^ or may never happen. This ongoing UCM may may lead to several unpleasant consequences for the married couple, such as divorce and infertility.^2^^,^^5^^,^^6^ The prevalence of UCM in the general population is unclear, and UCM is reported to account for up to 17% of visits to sexual health clinics,^7^ mainly in some conservative Middle-Eastern societies and in developing countries.^2^^,^^7-9^ Occurrences of female sexual dysfunction were previously deemed to be the major factors leading to UCM,^2^^,^^10^ but more recent studies have shown that the importance of problems in men in UCM have been underestimated in both Eastern and Western countries, such as Iran, Egypt, and Italy.^1^^,^^7^^,^^11^ According to literature data, vaginal penetration phobia (VPP) and vaginismus are the most common female sexual disorders in UCM couples,^7^^,^^9^^,^^11-14^ and premature ejaculation (PE) and erectile dysfunction (ED) account for the most frequent male sexual disorders.^2^^,^^4^^,^^9^^,^^15^^,^^16^

Many studies have demonstrated the role of religious, cultural, and psychological factors in the occurrence of UCM, such as lack of sufficient sexual information, misconception about genitalia, sexual performance anxiety, lack of privacy, history of sexual abuse and posttraumatic stress disorder (PTSD), sexual prohibitions applied by religion, family, and society, and also conventions associated with the first sexual intercourse after marriage.^17-19^ It is interesting that, so far, no data exist on the phenomenon of UCM in relation to China, the most populated country in the world and also a place where traditionalism and open attitudes interlace in a complex and fascinating manner.^20^ Since China’s cultural and economic expansion in 1978, rapid development and urbanization have taken place, and modernization and globalization have led to more open attitudes toward a wide range of social issues. Ideologies and conventions regarding sex ideologies are part of this evolving scenario, but attitudes toward sex seem to be, at least in some regions of the Chinese continent, more conservative than those of Western countries.^21^^,^^22^ These relatively conservative attitudes are particularly evident people from rural areas,^23^ where there is a lack of reliable information about sex and traditional approaches and beliefs may exert a greater influence.^24^^,^^25^ Therefore, it is reasonable to suppose that a degree of conservatism in attitude toward sex may contribute to the occurrence of UCM in Chinese couples, as well as to specific practices that may with respect to those of other countries where this phenomenon occurs and has already been reported. Hence, we conducted this study to retrospectively investigate the clinical characteristics and treatment outcomes among Chinese couples with UCM who presented for clinical consultation to the Department of Infertility and Sexual Medicine at the Third Affiliated Hospital of Sun Yat-sen University, to collect preliminary data regarding the occurrence of this interesting phenomenon in a Chinese milieu.

## Material and methods

Data were collected on 127 couples with UCM who were evaluated and treated in outpatient clinics from January 2019 to May 2021. Due to the lack of clear diagnostic criteria and dedicated guidelines for this disorder, the diagnostic criterion for UCM for this study was defined as the failure of penovaginal penetration despite repeated attempts (at least 2 attempts).^12^ Data regarding evaluation and treatment strategies were collected retrospectively. The protocol and written informed consent used in this study were reviewed and approved by the Institutional Review Board, trial registration number: (2019)02-541-01.

The patients were evaluated and counseled by relevant specialists, including andrologists for male patients, gynecologists for female patients, and sex therapists (women who were systematically trained by the Chinese Sexology Association) for both. The marital partners were first consulted separately and then together to analyze the etiology more accurately and further provide more effective treatment options. Both partners were first asked about their medical, sexual, psychosexual, and personal history and then underwent physical examinations.

### Female evaluation

All female patients were asked to undergo gynecological examinations for assessment of vaginal trophism and the condition of the hymen. Despite the existence of specific criteria for the diagnosis of genitopelvic pain/penetration disorder according to the 5th edition of the *Diagnostic and Statistical Manual of Mental Disorders* (DSM-5) ^26^ of the American Psychiatric Association, we preferred to adopt the older criteria of DSM-IV^27^ to evaluate vaginismus and dyspareunia separately. Diagnostic criteria for dyspareunia are the following: 1. Recurrent or persistent genital pain associated with sexual intercourse. 2. The disturbance causes marked distress or interpersonal difficulty (e.g. avoidance of sexual experiences, disrupting existing sexual relationships). 3. The disturbance is not caused exclusively by lack of lubrication and is not due exclusively to the direct physiological effects of a substance (e.g. a drug of abuse or medication) or a general medical condition. The diagnostic criteria for vaginismus are the following: 1. Recurrent or persistent involuntary spasm of the musculature of the outer third of the vagina that interferes with sexual intercourse. 2. The disturbance causes marked distress or interpersonal difficulty, but (sexual responses such as desire, pleasure, and orgasmic capacity) may not be impaired. 3. The disturbance is not due exclusively to the direct physiological effects of a general medical condition.

### Male evaluation

All male patients were asked to undergo a uroandrological examination, including the levels of testosterone, prolactin, follicle-stimulating hormone (FSH), and luteinizing hormone (LH). If the patients complained of ED, they also underwent an audiovisual sexual stimulation (AVSS) test and/or a nocturnal penile tumescence and rigidity (NPTR) test with Rigiscan to exclude the possibility of organic ED. The diagnostic criteria to exclude ED with Rigiscan were penile tip rigidity more than 60% and minimum duration of 10 minutes (NPTR)^17^/9 minutes (AVSS).^28^ The erection hardness score (EHS)^29^ and the masturbation erection index (MEI)^30^ were used to assist in determining erectile function. Finally, by asking men if the ejaculation occurred before vaginal insertion with light or none touch stimulus, the presence of ejaculation ante portas was assessed.

### Combined evaluation

The couples proceeded with sex therapy in the treatment room. The female therapist conducted an illustrated explanation about the anatomy and physiology of genital organs, sexual response cycle, and misconceptions about sexual behaviors. For female patients who had VPP and vaginismus, once their anxiety and emotional tension had been reduced, they underwent desensitization by receiving vaginal dilation training through the gradual insertion of a finger or dildo into the vagina. Male patients were asked to learn about vaginal dilation treatment to let them know the right position of the vaginal orifice and the ability of the vagina to expand and contract, thereby boosting their sexual confidence. Meanwhile, female patients learned to develop a sense of security with their partner during sexual intercourse.

### Informing couples (and their families, if necessary)

Patients were both informed about the condition of the partner, in order to help them to address the problem by sharing a couple strategy. Meanwhile, for couples who visited our department with their family members, therapists were responsible for explaining the condition of UCM to the families of the patients because, from a Chinese cultural standpoint, family and parental distress deriving from UCM (often correlated with inability to conceive^31^) often worsens the distress already perceived by the couple.

### Therapeutic workup

The husband was taught to use 1 finger first to penetrate the partner’s vagina in order to get the vagina acclimated under the guidance of therapist. Then, he tried to enter with 2 fingers, and this step could have taken time for some females with moderate-to-severe vaginismus. Once the vagina was able to accommodate 2 fingers, the couple could attempt to have vaginal intercourse.

For couples who agreed to have sexual intercourse in the hospital, the female patients first had vaginal dilation with a dildo. Meanwhile, the male patients with ED were prescribed Tadalafil (10-20 mg) orally 2 hours before intercourse to ensure a penile erection; in case of PE, patients were instructed by the therapists to take 30 mg dapoxetine plus 10-20 mg tadalafil 2 hours before intercourse. Then, the couples tried to have sexual intercourse in a man-on-top position. A lubricating agent was provided to assist in penile penetration of the vagina. The therapists left the room after providing the instructions, and the couple had sexual intercourse privately.

For the couples who did not agree to have sexual intercourse in the hospital, the husband were asked to dilate the vagina with fingers at home before intercourse to make his partner feel prepared and secure physically and mentally. The female therapist then instructed the couple to have sexual intercourse in a man-on-top position. The male patients were asked to take 5 mg tadalafil orally once a day for 5-7 days before intercourse. All couples were followed up online every month and asked to come back to the hospital if necessary.

### Statistical analysis

Continuous variables were represented as means and standard deviations. Categorical variables were represented as absolute and percentage frequencies.

## Results

All couples stated not to have any extramarital sexual activity. Demographic data for the couples are shown in Table 1. Among these couples, 93 couples (73.2%) visited the andrologist first, and 34 couples (26.8%) visited the gynecologist first. The mean ages of the male and female patients were 33.1 (7.0) years (range 25-39, median 25 years) and 29.5 (4.1) years (range 23-37, median 30 years), respectively. The average marriage duration was 32.7 months.

**Table 1:** Demographic information.

**Variable**	**Study participants**	
	**Males**	**Females**
Total no. of participants	127	127
Age, years, mean (SD)	33.1 (7.0)	29.5 (4.1)
BMI, mean (SD)	23.8 (3.7)	20.7 (2.6)
Educational background, No. (%) of participants		
College degree or higher	64 (50.4%)	60 (47.2%)
Technical secondary school	31 (24.4%)	35 (27.6%)
High school or below	32 (25.2%)	32 (25.2%)
Marriage duration, months, mean (SD)	32.7 (24.9)	

In this study, 64 men (50.4%) and 60 women (47.2%) had a college degree or higher. The etiological factors of UCM found in this study are listed in Table 2. Among 127 couples, UCM was caused either by male factors alone, such as ED (32, 14.7%), PE (1, 0.5%), ED/PE (2, 0.9%), and low sexual desire (1, 0.5%); female factors alone, such as vaginismus (21, 9.7%), dyspareunia (17, 7.8%), and vaginismus/dyspareunia (17, 7.8%), or a combination of male and female factors (36 couples, 16.6%). Among 64 patients diagnosed with ED, 56 men were identified as having nonorganic ED and 8 were suspected to have organic ED and needed further evaluation. When asked about personal history, female patients often reported strict control by parents (28, 30.8%), hearing about unpleasant sexual experiences from friends (28, 30.8%), having a sense of insecurity (14, 15.4%), and having a painful experience during the first attempt at intercourse (15, 16.5%). Three female patients admitted that they had acrophobia, hemophobia, and trypanophobia, respectively. Female patients acuried information about sex from the internet (37, 40.7%), friends (34, 37.7%), husband or boyfriend (24, 26.4%), parents (22, 24.2%), and adult videos (21, 23.1%).

**Table 2:** Etiological factors of unconsummated marriage.

	**UCM Couples**	
	**No.**	**%**
Female factors only		
Total	55	25.3%
Vaginismus	21	9.7%
Dyspareunia	17	7.8%
Vaginismus with dyspareunia	17	7.8%
Male factors only	36	16.6%
ED	32	14.7%
PE	1	0.5%
ED with PE	2	0.9%
Low sexual desire	1	0.5%
Male and female factors		
Total	36	16.6%
Vaginismus/ED	18	8.3%
Vaginismus/PE	1	0.5%
Vaginismus with dyspareunia/ED	8	3.7%
Vaginismus with dyspareunia/PE	2	0.9%
Dyspareunia/ED	4	1.8%
Dyspareunia/PE	1	0.5%
Lack of sexual knowledge	2	0.9%
Total	127	58.5%

**Abbreviations:** ED, erectile dysfunction; PE, premature ejaculation; UCM, unconsummated marriage.

A total of 71 couples (55.9%) stated that they were uncertain about the etiology of their UCM. Twenty eight couples (22.0%) believed that penetration failure was due to the male partner, whereas 28 (22.0%) believed only the female had a problem. The self-perceived etiology of each patient was recorded and compared with the doctor’s diagnosis. Self-reports of 12 couples (9.4%) did not correspond to the final doctor’s diagnosis, including 10 women belonging to couples where UCM was spontaneously attributed to the male partner, were instead diagnosed with female sexual dysfunction (FSD), whereas 2 men were unexpectedly diagnosed with ED although the UCM was reported at first to be related to the women.

After treatment and guidance by doctors and therapists, a total of 86 couples were able to have intercourse successfully (success rate 67.7%). In addition, at the time of the draft of the present manuscript, 40 couples, were still under treatment and were being followed up, and 1 couple had divorced.

## Discussion

From January 2019 to May 2021This study examined 127 consecutive couples in unconsummated marriage from. Unconsummated marriage among these Chinese couples was predominantly caused by female factors (55.8%). Two primary factors contributed to inability to achieve a penovaginal penetration: genitopelvic pain/penetration disorder (female) and ED (male).

Differently from Middle Eastern and Western countries,^7^^,^^14^^,^^32^ in China women’s attitudes and cognitions toward sexuality are often influenced by their parents and female friends instead of religious reasons.^33^ Negative information about sex conveyed by parents and friends causes fear of sex in some young women, thereby influencing their sexual function. So far, there has been no formal sexual health education in schools in China, and young people often learn about sex from their peers or videos on the internet.^34^ Sexual re-education by a trained specialist, with teaching about genital anatomy and physiology, may prove particularly useful in these cases.

The mean duration of UCM in our study, was 32.7 months, which is shorter than durations reported for some Western studies.^1^ This difference may occur because in China, couples with UCM are often pressed by their family to an immediate medical consultation to solve the problem. Indeed, procreation is a relevant issue in China, especially in the eyes of older generations.^20^^,^^31^^,^^35^^,^^36^ When it comes to infertility, patients, especially men, often come to the hospital for consultation under the pressure of their families. First, they usually seek treatment for UCM in andrology or gynecology clinics due to their limited knowledge about what a “couple dysfunction,” as UCM should be considered, really is, but also due to the fact that there are very few centers for couple sexual health in China. Meanwhile, due to cultural and social limitations, people express pudency and cannot fully disclose their problems when talking about sex, even to doctors. The risk of missed diagnoses may be increased because the specialists usually make a diagnosis merely based on the evaluation of 1 partner in a couple rather than both the male and female partners,^37^ which is the reason why for this study excluded patients who came for clinical consultation alone rather than as a couple. Also, as the results show, there is often a discrepancy between the patients’ description and the actual situation of the couple, which motivates the need for a proper re-education, on the part of the sex therapist, of patient expectations about sexual intercourse. Regarding organic problems, we need to consider that erection is a complex neurovascular phenomenon involving psychogenic, neurologic, vascular, and cavernosal factors.^38^^,^^39^ On one hand, repeated penetration failure, eg, vaginismus, may lead to secondary ED,^7^^,^^10^^,^^12^^,^^32^ and hence medical therapy with phosphodiesterase type 5 inhibitor (PDE-5i) may be particularly useful to give confidence to the man in attempting penovaginal penetration. On the other hand, without comprehensive diagnosis and psychosexological counseling, drug therapy alone may be likely o aggravate the woman’s FSD and further reduce the man’s confidence. We suggest, when a patient complains that his erection is “not firm enough,” that the doctor should ask specifically whether he has completed at least 1 full vaginal penetration before. This detail is a very important differential diagnostic clue, which can distinguish between generalized ED and UCM with high accuracy and should prompt further investigation. However, UCM refers to the failure to perform sexual intercourse by a couple rather than an individual in the couple; therefore, if a couple is diagnosed with UCM, both husband and wife should be treated individually as well as guided by sex therapists toward successful sexual intercourse.

There are some limitations to this study. Ultrasonography was not performed to determine the cause of organic ED, while females could not perform pelvic ultrasound nor hormonal analysis. Moreover, patients complaining about UCM who came to consultation alone, and not as a couple, were not included in the statistics.

## Conclusion

Unconsummated marriage among Chinese couples is a common phenomenon; however, data about its prevalence and characteristics are lacking. With this study, we first demonstrated that UCM can be caused by male and female factors individually or in combination, but the presence of a female factor is predominant. Vaginismus and ED are the main conditions, and lack of sexual knowledge as well as cultural beliefs play important roles. Further research should be directed toward establishing the real prevalence of this phenomenon in China, as well as regional diversities, which may influence the incidence rates and treatment outcomes. In any case, for couples with UCM, separate evaluation by andrologists and gynecologists, with a detailed assessment of psychosexual history followed by combined evaluation and treatment conducted by a trained sex therapist, remains the most effective workup for the treatment of this couple dysfunction.

## Funding

This work was supported by the National Natural Science Foundation of China (grant nos. 81571424 and 81771565).

*Conflicts of interest:* None declared.
