# Correction to: Unconsummated marriage among Chinese couples: a retrospective study

**DOI:** 10.1093/sexmed/qfag046

**Published:** 2026-06-08

This is a **correction** to: Yu Xi, Tingting Xia, Elena Colonnello, Chunlin Wang, Yufen Lai, Yan Zhang, Unconsummated marriage among Chinese couples: a retrospective study, *Sexual Medicine*, Volume 11, Issue 1, February 2023, qfac003, https://doi.org/10.1093/sexmed/qfac003

In the originally published version of this manuscript, the percentage values in Table 2 were incorrect. The corrected Table 2 is reproduced below:


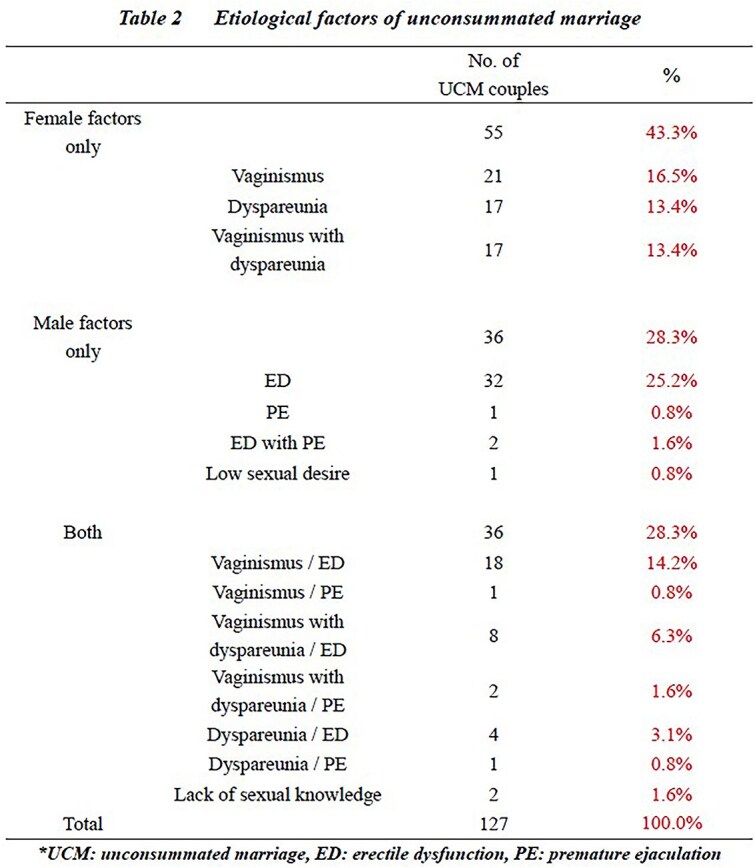


As a result of these updates, the second sentence of the second paragraph in the Results section should now be read as following:

``Among 127 couples, UCM was caused either by male factors alone [such as ED (32, 25.2%), PE (1, 0.8%), ED with PE (2, 1.6%), and low sexual desire (1, 0.8%)], female factors alone [such as vaginismus (21, 16.5%), dyspareunia (17, 13.4%), and vaginismus/dyspareunia (17, 13.4%)], or a combination of male and female factors (36 couples, 28.3%).''

These changes do not alter the study's overall findings or conclusion.

These details have been corrected only in this **correction notice** to preserve the published version of record.

